# μ-Opioid Receptors on Distinct Neuronal Populations Mediate Different Aspects of Opioid Reward-Related Behaviors

**DOI:** 10.1523/ENEURO.0146-20.2020

**Published:** 2020-09-18

**Authors:** Amie L. Severino, Nitish Mittal, Joshua K. Hakimian, Nathanial Velarde, Ani Minasyan, Ralph Albert, Carlos Torres, Nicole Romaneschi, Camille Johnston, Suchi Tiwari, Alex S. Lee, Anna M. Taylor, Claire Gavériaux-Ruff, Brigitte L. Kieffer, Christopher J. Evans, Catherine M. Cahill, Wendy M. Walwyn

**Affiliations:** 1 Department of Psychiatry and Biobehavioral Sciences, UCLA Semel Institute for Neuroscience and Human Behavior, David Geffen School of Medicine, University of California Los Angeles, Los Angeles, CA 90095; 2 Division of Pharmacology and Toxicology, College of Pharmacy, University of Texas at Austin, Austin, TX 7871; 3 ZS Associates, San Mateo, CA 94402; 4 Department of Pharmacology, University of Alberta, Edmonton, Alberta,T6G 2R3 Canada; 5 UCLA Brain Research Institute, University of California Los Angeles, Los Angeles, CA 90095; 6 Department of Translational Medicine and Neurogenetics, Institut de Génétique et de Biologie Moléculaire et Cellulaire, Illkirch, France, UM7104; 7 Université de Strasbourg, Illkirch, France, 67081; 8 Centre National de la Recherche Scientifique, Unité Mixte de Recherche 7104, Illkirch, France; 9 Institut National de la Santé et de la Recherche Médicale, Unité 964, Illkirch, France; 10 Ecole Supérieure de Biotechnologie de Strasbourg, Illkirch, France, CS10413; 11 Department of Psychiatry, Douglas Mental Health Institute, McGill University, Montreal, Quebec, QC H4H 1R3 Canada

**Keywords:** floxed MOR, hyperlocomotion, intravenous self-administration, morphine, μ-opioid receptor, oxycodone

## Abstract

μ-Opioid receptors (MORs) are densely expressed in different brain regions known to mediate reward. One such region is the striatum where MORs are densely expressed, yet the role of these MOR populations in modulating reward is relatively unknown. We have begun to address this question by using a series of genetically engineered mice based on the Cre recombinase/loxP system to selectively delete MORs from specific neurons enriched in the striatum: dopamine 1 (D1) receptors, D2 receptors, adenosine 2a (A2a) receptors, and choline acetyltransferase (ChAT). We first determined the effects of each deletion on opioid-induced locomotion, a striatal and dopamine-dependent behavior. We show that MOR deletion from D1 neurons reduced opioid (morphine and oxycodone)-induced hyperlocomotion, whereas deleting MORs from A2a neurons resulted in enhanced opioid-induced locomotion, and deleting MORs from D2 or ChAT neurons had no effect. We also present the effect of each deletion on opioid intravenous self-administration. We first assessed the acquisition of this behavior using remifentanil as the reinforcing opioid and found no effect of genotype. Mice were then transitioned to oxycodone as the reinforcer and maintained here for 9 d. Again, no genotype effect was found. However, when mice underwent 3 d of extinction training, during which the drug was not delivered, but all cues remained as during the maintenance phase, drug-seeking behavior was enhanced when MORs were deleted from A2a or ChAT neurons. These findings show that these selective MOR populations play specific roles in reward-associated behaviors.

## Significance Statement

μ-Opioid receptors (MORs) mediate the effects of the commonly misused and prescribed opioids. These receptors are expressed in different neurons and pathways mediating reward. Although it is well known that μ receptors in the midbrain regulate dopamine release and are important in mediating reward, little is known of the role of other populations that are expressed in the different neurons of the striatum, a hub of many reward pathways. In this study, we deleted selective populations of these receptors that are enriched in the striatum and studied the effect of each deletion on reward-related behaviors. We found that each population plays a specific role in reward demonstrating a more complex role than previously thought of how these receptors mediate reward.

## Introduction

μ-Opioid receptors (MORs), the principal target of addictive analgesics are widely expressed in diverse brain regions associated with reward (for review, see Le Merrer et al., 2009). MORs are expressed on the GABAergic neurons that innervate the dopaminergic neurons of the ventral tegmental area (VTA) so are poised to enable dopamine release (Ben Hamida et al., 2017; Charbogne et al., 2017) an important mediator of rewarding behavior. MORs are also expressed in the striatum which controls movement and the formation of behavioral habits associated with reward. These two behaviors, reward and locomotion, are mediated by different dopaminergic signaling profiles in distinct neurons (Howe and Dombeck, 2016) and are often used to generate a profile of reward behavior in mice (Mitchell et al., 2005; Zhang and Kong, 2017).

MORs are widely expressed in the different neuronal populations and subregions of the striatum (Wang et al., 1996, 1997; Wang and Pickel, 1998; Miura et al., 2008; Cui et al., 2014). They are expressed on dopamine 1 (D1) receptor, D2, and adenosine 2a (A2a) subpopulations of medium spiny neurons (Cui et al., 2014; Oude Ophuis et al., 2014). They are also expressed on cholinergic interneurons (Ponterio et al., 2013) and on cortical or thalamic glutamatergic neurons innervating medium spiny neurons (for review, see Miura et al., 2008). Within these different neuronal populations, MORs are differentially expressed in striatal subregions. For example, they are expressed in the patches or striasomes where they colocalize with dynorphin-expressing D1 medium spiny neurons (Brimblecombe and Cragg, 2017) but also in the matrix where their expression is less and on D1 or D2 medium spiny neurons (Cui et al., 2014).

Although we do not fully understand the functional role of each of the striatal neuronal populations, we do have insight as to their function from their cellular expression patterns and electrophysiology studies. MORs are expressed presynaptically on glutamatergic afferents projecting to the striatum and postsynaptically on striatal dendrites and dendritic spines (Wang et al., 1996). Activation of these receptors inhibits both glutamatergic afferent activity and that of GABAergic collaterals from medium spiny neurons (Blomeley and Bracci, 2011; Ma et al., 2012; James et al., 2013). In addition, MORs inhibit cholinergic interneurons so regulating local spontaneous dopamine release (Sandor et al., 1992; Ponterio et al., 2013; Ponterio et al., 2018). Presynaptic MORs are also found on low threshold spike interneurons and so modulate their spontaneous activity (Elghaba and Bracci, 2017). At the behavioral level, several studies point toward a role of striatal MOR populations in reward behaviors. Earlier studies showed that ablating MOR-enriched striosomes of the dorsal striatum produces deficits in motor-skill learning (Lawhorn et al., 2009). Forebrain MORs are known to play a role in alcohol, food and heroin reward behaviors (Ben Hamida et al., 2017; Charbogne et al., 2017) and in the hedonic reward value of food reward (Boulos et al., 2019). In addition, MOR re-expression on dynorphin expressing medium spiny neurons in an otherwise null background are sufficient to reinstate some, but not all, opioid reward behaviors (Cui et al., 2014).

Given the broad but diverse distribution of MORs on different neuronal subtypes throughout the striatum, we set out to determine the contribution of these MORs to opioid reward behaviors. In order to do this, we bred flMOR mice, in which exons two and three of the MOR gene (*oprm1*) are flanked by LoxP, with four different Cre-recombinase mice (D1cre, D2cre, A2acre, ChATcre). We first verified these deletions using RNAScope *in situ* mRNA hybridization and quantitative PCR. We then assessed opioid-induced hyperlocomotor, sensitization of this effect, and intravenous opioid self-administration (IVSA). From these studies, we conclude that each of these MOR-expressing populations are required for distinct aspects of opioid reward-related behaviors.

## Materials and Methods

### Experimental design

#### Subjects

All procedures were authorized by the Institutional Animal Care and Use Committee (IACUC) and are in compliance with the Policies on the Use of Animals in Research as outlined by this journal. All transgenic mice used in this study were bred by the Animal Breeding Colony. D1flMORs, D2flMORS, A2aflMORs and choline acetyltransferase (ChAT) flMORs were generated by breeding flMOR mice (loxP sites flanking exons 2–3 of the *oprm1* gene on a 50:50 C57BL/6J:129Sv background, stock #030074, The Jackson Laboratory) with four Cre driver lines to obtain Cre recombinase on one (D1cre; stock #030989-UCD, D2cre; 032108-UCD, A2acre; 036158-UCD, MMRRC, NIH, DHHS, 100% C57BL/6J) or two (ChAT-IRES-Cre; stock #028861, The Jackson Laboratory, 100% C57BL/6J) alleles and flMOR on both alleles. Control flMOR mice of the same background were generated as littermates from the breeding strategies used. Mice lacking all MORs (stock #007559, 100% C57BL/6J, The Jackson Laboratory) were bred as heterozygous pairs to generate knock-out (KO) and wild-type (WT) littermates. Male and female transgenic mice were used between age 8–32 weeks and 20–36 g of body weight. Animals were maintained on a 12/12 h light/dark cycle with *ad libitum* access to food and water, and experiments were conducted at ZT4–ZT8 (Zeitgeber Time). All mice were group housed for the duration of the experiment except for the IVSA experiments during which mice were singly housed in an enriched environment after surgery.

#### Compounds

All Schedule II drugs, remifentanil, oxycodone, cocaine, and morphine, were obtained from the NIDA Drug Supply Program (RTI).

#### RNA *in situ* hybridization and light sheet fluorescent microscopy

Mice were euthanized, their brains removed and flash frozen. All equipment and surfaces were cleaned with RNase inhibitor solution and ISH (Advanced Cell Diagnostics) performed as previously described (Severino et al., 2018). To characterize MOR knock-down in the D1-, D2-, A2a-, and -flMOR mice, the following riboprobes were used; *oprm1* (catalog #315841, Atto 550), *drd2* (catalog #406501-C2, Alexa Fluor 488), and *drd1a* (catalog #406491-C3, Atto 647). To characterize MOR knock-down in ChATflMOR mice, the same *oprm1* and *drd1a* riboprobes were used as well as a *ChAT* riboprobe (catalog #408731-C2, Alexa Fluor 488). RNA *in situ* hybridization was imaged using a 63× oil immersion objective on a Leica SP8 stimulated emission depletion microscope (STED, Leica Microsystems) at the Advanced Light Microscopy Core. The images were compiled in Adobe Illustrator 2019 and brightness and contrast and the tonal adjustments feature uniformly applied across the entire composite image. To determine the extent to which MOR was deleted from specific neuronal types within each of the mouse lines generated, we counted the number of *drd1a*, *drd2*, or *ChAT-*positive cells and then determined the number of these cells that were MOR positive (having a minimum of three grains). The data are expressed as the percentage of MOR-positive cells within each of the subgroups (*drd1a*, *drd2*, or *ChAT*).

#### Quantitative reverse transcription-PCR (qPCR)

qPCR was performed in flMOR, D1-, D2-, A2a-, and ChAT-flMOR mice to define the relative expression levels of *oprm1*, *drd1a*, and *drd2* using the primers shown in Table 1 and methodology as previously described (Hakimian et al., 2017). Relative ratios comparing conditional KOs to flMOR expression for each gene of interest were calculated by using β-actin as reference gene and the 2^-ΔΔCt^ method to evaluate differential expression levels.

**Table 1:** The primer sequences used in the qPCR validation of MOR knock-down

Gene primer sequences (5’−3’)	
OPRM1 FWD	TCAAGGCCCTGGATTTCCGTACCC
OPRM1 RVS	CGGGCAGACCAATGGCAGAAGAGA
DRD1 FWD	CTTGTCTGTGCCGCTGTCATCAGG
DRD1 RVS	GGCATGACCAAGACAGCCACCAAG
DRD2 FWD	TTGTTCTTGGTGTGTTCATC
DRD2 RVS	TATAGATGATGGGGTTCACG
ACTB FWD	TGTGCACTTTTATTGGTCTC
ACTB RVS	GATGTATGAAGGCTTTGGTC

OPRM1; μ-opioid receptor, DRD1; dopamine 1 receptor, DRD2; dopamine 2 receptor, ACTB; β-actin control, FWD; forward, RVS; reverse.

#### Open-field locomotion

Fiberglass open field boxes (28 × 28 × 18 cm) were placed on a horizontal glass pane 71 cm above an infrared camera (acA1300-60gm Basler ace camera) at 250 lux. After 2 d of habituation, mice were placed in the chamber for 15 min followed by a subcutaneous injection of saline or drug and placed back in the chamber for 60 min and their locomotion activity recorded (Ethovision XT10, Noldus). This was repeated at the same time of day for three consecutive days.

#### IVSA

An intravenous catheter (0.2 mm i.d., 0.4 mm o.d., Norfolk Access) was inserted into the right jugular vein of mice under sterile conditions as previously described (James et al., 2013; Storey et al., 2016; Mittal et al., 2017). After 3 d of recovery, the mice began daily self-administration in operant chambers (Med-Associates) for 2 h or 50 reinforcers, whichever came sooner. A two-lever design was used in which the active cue and drug-paired lever, or the inactive lever, was randomly assigned. An active lever press resulted in an intravenous drug infusion (0.67 μl/g body weight) and the presentation of a 10-s tone and visual light cue. Each reinforcer was followed by a 10-s “timeout” period during which no reinforcers could be delivered but presses could be made on either lever. On the first 2 d of this protocol, mouse exploration of the levers was facilitated by placing a drop of 20% sweetened condensed milk on both the active and inactive levers (3× per session). The mice initially underwent 3–5 d of acquisition training using remifentanil (0.05 mg/kg/infusion) at a fixed ratio of one (one lever press resulted in one infusion, FR1). Oxycodone (0.25 mg/kg/infusion) was then used as the reinforcer for nine consecutive days, the maintenance phase, on the same FR1 schedule. This was followed by extinction training over 3 d during which the mice underwent the same FR1 schedule to a maximum of 50 reinforcers or 2 h, but saline was delivered through the catheter. Catheter patency was tested using an infusion of propofol (20 μl of 1% propofol w/v in saline) every 5 d.

### Statistical analysis

Power analyses of prior data indicate that power is 0.8 or greater with cell means of *n* = 8 (*n* = 12 used for experiments where animal drop-out rates are expected because of jugular cannula failure, etc.). For experiments where we lacked sufficient prior data for an a priori power analysis, we used prior experience with similar methods to guide us. Although we did use male and female mice, we did not analyze sex as a biological factor as we did not have sufficient power to do so. All experiments included both genotypes with males and females representing 46% and 53%, respectively, of the total number of mice used.

### Several analytical methods were used

#### ANOVA

One-way or two-way ANOVA was used to analyze data obtained from the RNA ISH, qPCR, total locomotion and the intrasession IVSA datasets using Prizm v8 (GraphPad) with further details provided in the results and statistical tables.

#### Linear mixed models (LMM)

LMM were used to analyze the intrasession locomotion data so as to examine the slope and so rate of change over time of this dataset. We also used LMM with coefficients accounting for random slope or intercept within subjects to define and interpret the intersession IVSA datasets. We used the lmerTest (Kuznetsova et al., 2017) package in R to run LMM. The linear models were used to assess the effect of time, treatment group, or an interaction of these factors on each variable. The resulting model is a regression equation where the intercept or the slope is allowed to vary for each subject:

$$Y_{Characteristic}=\;\beta_{0}+\;\beta_{Group}X_{Group}*\;\beta_{Day}X_{Day}+\;U_{Subject},$$

where Y_Characteristic_ is the characteristic being modeled (e.g., distance traveled, lever presses, etc.), each predictor variable is represented by its subscripted X, U_Subject_ represents the random intercept or slope associated with each individual subject. The coefficients (β) are estimated and assessed for significance. Whenever a significant effect was observed, an ANOVA against a reduced null model was used to assess the impact of the respective factor.

## Results

### Validation of the selectivity and extent of MOR knock-down in striatal subpopulations

**Figure 1.**
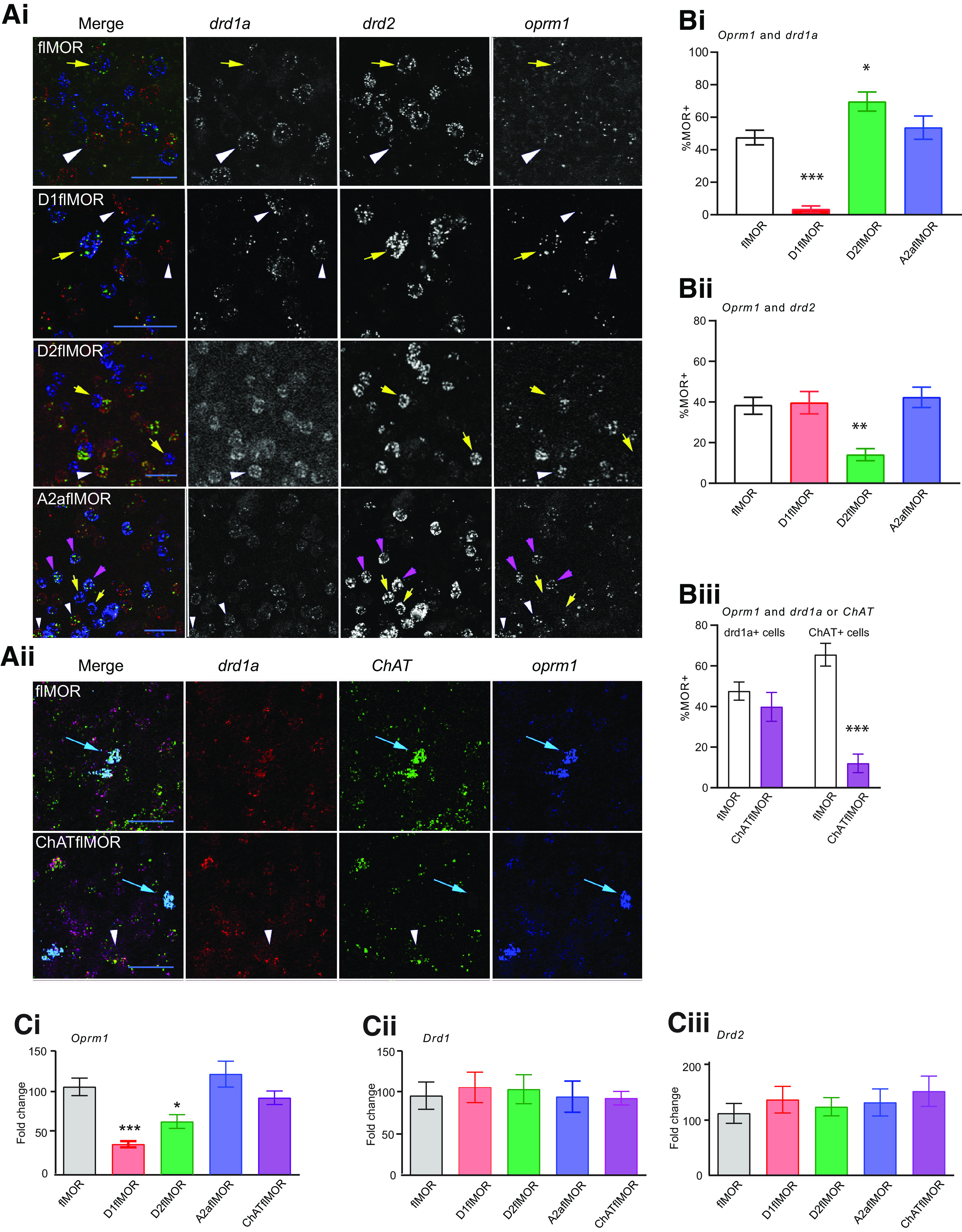
Validation of the selectivity and extent of MOR knock-down in striatal subpopulations. ***Ai***, Representative RNA *in situ* hybridization images for MOR (*oprm1* in white), dopamine receptor 1 (D1 or *drd1a* in red) and dopamine receptor 2 (D2 or *drd2* in green), are shown from the dorsolateral striatum of control, flMOR, and D1flMOR, D2flMOR, and A2aflMOR mouse lines. White arrows without a tail demonstrate D1-expressing cells and yellow arrows with a tail show D2-expressing cells. The cells marked by pink arrows in the A2aflMOR images show cells that are *oprm1* and *drd2* positive. ***Aii***, Representative *RNA in situ* hybridization images of the dorsolateral striatum showing *oprm1* (white), ChAT (green), and D1 (red) labeling in flMOR and ChATflMOR lines. Arrows highlight ChAT+ cells. Scale bar = 20 μm (***Ai***, ***Aii***). ***B***, *Oprm1* expression were quantified and presented as the % colocalization for each genotype of MOR with D1+ cells in ***Bi***, MOR with D2+ cells in ***Bii***, and MOR with ChAT+ or D1+ cells in ***Bii***; *p < 0.05, **p < 0.01, and ***p < 0.001 versus flMORs of the same experiment. ***C***, qPCR for *oprm1*, *drd1*, and *drd2* cDNA levels in striatal tissue shows reduced *oprm1*expression in D1flMORs (***p < 0.001) and D2flMORs (*p < 0.05) compared with flMORs. There was no effect of these MOR deletions on *drd1* (***Cii***) or *drd2* (***Ciii***) expression. Refer to Table 2 for statistical analyses. All data are shown as mean ± SEM, and the individual datapoints are shown in Extended Data Figure 1-1, for which this legend also applies.

We first defined the selectivity of the loxP/Cre recombinase system by RNA *in situ* hybridization to examine cell-specific knock-down of the MOR encoding gene (*oprm1*) in the dorsolateral striatum. We found that, for cells labeled with the *drd1* probe, *oprm1* and *drd1* colocalization was reduced in D1flMORs (representative image, Fig. 1*Ai*; quantified expression, Fig. 1*Bi*; *p* < 0.001, Table 2, item a) and enhanced in D2flMORs (Fig. 1*Bi*; *p* < 0.05, Table 2, item a). For cells labeled by the *drd2* probe, *oprm1* and *drd2* colocalization was reduced in D2flMORs (representative image, Fig. 1*Ai*; quantified expression, Fig. 1*Bii*; *p* < 0.01, Table 2, item b). A2aflMORs showed *oprm1* expression in *drd1*+ cells and a deletion from some, but not all *drd2+* cells (representative image, Fig. 1*Ai*; quantified expression, Fig. 1*Bii*; N.S, Table 2, item b). In assessing *oprm1* expression in ChAT+ cells, we found a loss of *oprm1* expression in ChATflMORs compared with flMORs but no change in *drd1* expression, a positive control (representative image, Fig. 1*Aii*; quantified expression, Fig. 1*Biii*; *p* < 0.0001, Table 2, item c).

**Table 2:** Statistical analyses of MOR knock-down in D1-, D2-, A2a-, and ChAT-flMOR lines by RNA ISH and qPCR (**Fig. 1)**

Item	Figure	Experiment	Statistical test	Effect or interaction	Main effect	flMOR	D1flMOR	D2flMOR	A2aflMOR	ChATflMOR
**a**	1*Bi*	RNA *in situ* hybridization	One-way ANOVA	Genotype, *oprm1* and *drd1* probes	*F*_(3,16)_ = 29.14, *p* < 0.0001	Reference genotype *n* = 5	*p* < 0.0001 *n* = 5	*p* = 0.02 *n* = 5	N.S. *n* = 5	N.A.
**b**	1*Bii*	RNA *in situ* hybridization	One-way ANOVA	Genotype, *oprm1* and *drd2* probes	*F*_(3,15)_ = 8.76, *p* = 0.0013	Reference genotype *n* = 5	*p* < 0.99 *n* = 5	*p* = 0.004 *n* = 5	N.S. *n* = 4	N.A.
**c**	1*Bii*	RNA *in situ* hybridization	One-way ANOVA	Genotype, *oprm1*, *drd1* and ChAT probes	*F*_(3,13)_ = 15.95, *p* = 0.0001	Reference genotype *n* = 4	N.A.	N.A.	N.A.	ChAT+ vs -; *p* < 0.0001, *n* = 4 D1+ vs -; NS *n* = 5 and 4
**d**	1*Ci*	qPCR	One-way ANOVA	Genotype, *oprm1* probe	*F*_(4,35)_ = 8.59, *p* < 0.0001	Reference genotype *n* = 13	*p* = 0.0002, *n* = 6	*p* = 0.015, *n* = 8	N.S., *n* = 6	N.S., *n* = 7
**e**	1*Cii*	qPCR	One-way ANOVA	Genotype, *drd1* probe	*F*_(6,26)_ = 0.339, N.S.	Reference genotype *n* = 6	N.S., *n* = 6	N.S., *n* = 5	N.S., *n* = 5	N.S., *n* = 6
**f**	1*Ciii*	qPCR	One-way ANOVA	Genotype, *drd2* probe	*F*_(5,29)_ = 0.925, N.S.	Reference genotype *n* = 10	N.S., *n* = 5	N.S., *n* = 7	N.S., *n* = 5	N.S., *n* = 6

The RNA probes used were; *oprm1* (MOR), *drd1* (D1 receptor), *drd2* (D2 receptor), and *ChAT* (cholineacetyltransferase) in flMOR, D1-, D2-, A2a-, and ChAT-flMOR lines. The qPCR probes used were *oprm1* (MOR), *drd1* (D1 receptor), *drd2* (D2 receptor) in flMOR, D1-, D2-, A2a-, and ChAT-flMOR lines. RNA ISH: RNA *in situ* hybridization, N.S.: not significant, N.A: not applicable

10.1523/ENEURO.0146-20.2020.f1-1 Extended Data Figure 1-1 *Validation of the selectivity and extent of MOR knockdown in striatal subpopulations.* **ai.** Representative *RNA in situ* hybridization images for MOR (*oprm1* in white), dopamine receptor 1 (D1 or *drd1a* in red) and dopamine receptor 2 (D2 or *drd2* in green), are shown from the dorsolateral striatum of control, flMOR, and D1flMOR, D2flMOR, and A2aflMOR mouse lines. White arrows without a tail demonstrate D1-expressing cells and yellow arrows with a tail show D2-expressing cells. The cells marked by pink arrows in the A2aflMOR images show cells that are *oprm1* and *drd2* positive. **aii.** Representative *RNA in situ* hybridization images of the dorsolateral striatum showing *oprm1* (white), ChAT (green) and D1 (red) labeling in flMOR and ChATflMOR lines. Arrows highlight ChAT+ cells. Scalebar=20µM for both i and ii. **b.** *Oprm1* expression were quantified and presented as the % colocalization for each genotype of MOR with D1+ cells in **bi**, MOR with D2+ cells in **bii**, and MOR with ChAT+ or D1+ cells in **biii**. *, **, *** p<0.05, 0.01 and 0.001 vs flMORs of the same experiment. **c**. qPCR for *oprm1*, *drd1*, and *drd2* cDNA levels in striatal tissue shows reduced *oprm1*expression in D1flMORs (***; p<0.001) and D2flMORs (*; p<0.05) compared with flMORs. There was no effect of these MOR deletions on *drd1* (ii) or *drd2* (iii) expression. Refer to Table 2 for statistical analyses. All data are shown as mean ± SEM with the individual datapoints included. Download Extended Data Figure 1-1, EPS file.

qPCR was performed to determine overall striatal expression levels of *oprm1*, *drd1*, and *drd2* in flMOR in the conditional knock-down strains. We found a loss of *oprm1* cDNA in D1flMORs (*p* = 0.0001) and D2flMORs (*p* = 0.015; Fig. 1*Ci*; Table 2, item d) but no other line. There was no compensatory effect of these MOR deletions on *drd1* (Fig. 1*Cii*; Table 2, item e) or *drd2* (Fig. 1*Ciii*; Table 2, item f) expression in the different lines.

### Selective MOR deletions define specific roles of D1 and A2a MOR populations in opioid-induced hyperlocomotion

#### Oxycodone

**Figure 2.**
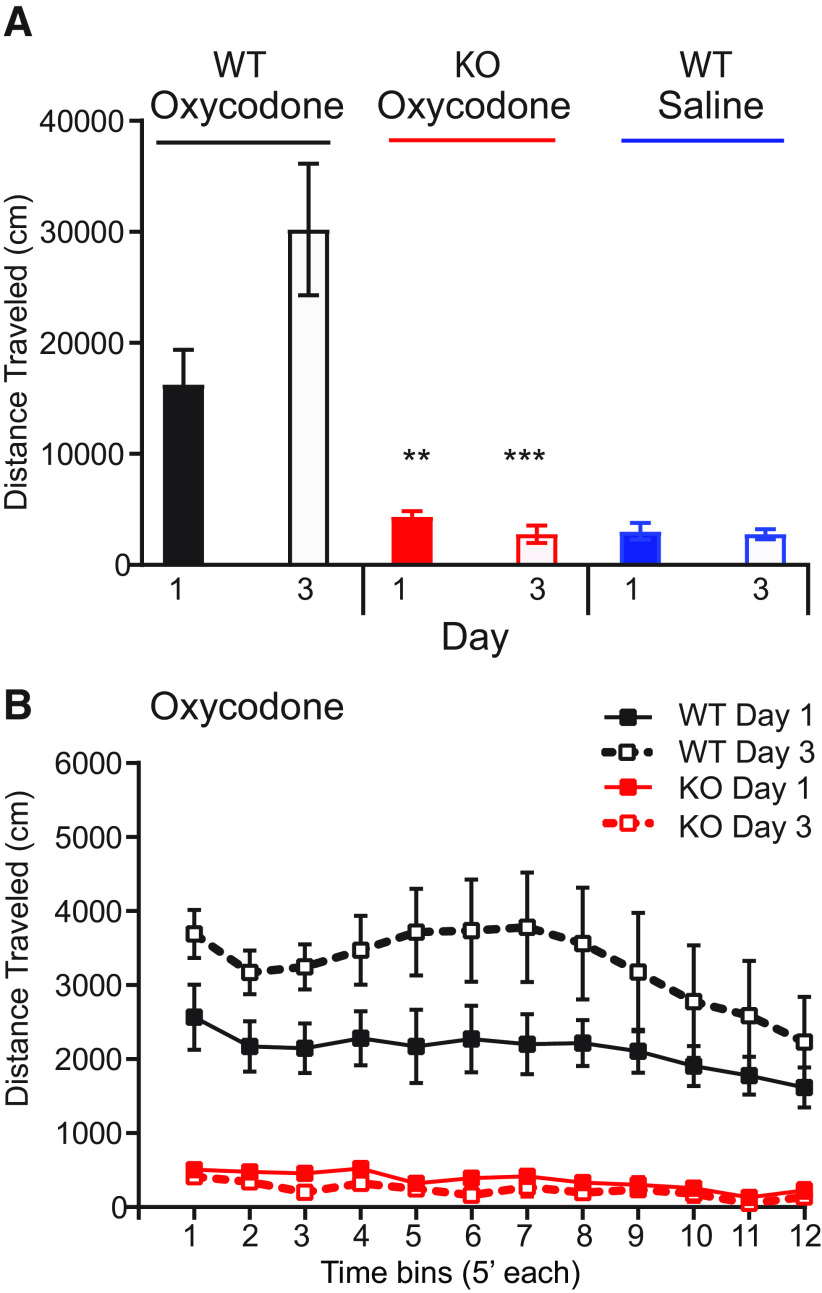
Oxycodone-induced locomotion is absent in constitutive MOR KOs. ***A***, Oxycodone (10 mg/kg, s.c.) induces hyperlocomotion in WT mice (WT) that is absent in mice lacking all MORs (KO) on the first (**p < 0.01 vs WT) and third (***p < 0.001 vs WT) days of three consecutive days of oxycodone. There was no difference between the effect of oxycodone in KO mice with that of saline in WT mice (*p* = 0.92). ***B***, The intrasession data (shown here in 5-min timebins for the 60-min test) further show the lack of effect of oxycodone in KO mice on days 1 and 3 (*p* < 0.0001 vs WT at all timepoints for both days). WTs demonstrated a sensitization of this locomotor response (*p* < 0.01) from day 1 to day 3 that was absent in KOs. Refer to Table 3 for statistical analyses. All data are shown as mean ± SEM, and the individual datapoints are shown in Extended Data Figure 2-1 for which this legend also applies.

As the analgesic effects of oxycodone may be non-specific (Yang et al., 2016), we first examined the locomotor effect of oxycodone (10 mg/kg, s.c.) in mice lacking MORs in all cells, a global MOR KO, and their WT littermates, (Fig. 2*A*) over three consecutive days. On day 1, we found no effect of oxycodone in MOR KOs compared with WTs (*p* < 0.01), a lack of effect that did not differ from WTs injected with saline (*p* = 0.92, Table 3, item a). By the third day, the oxycodone locomotor response had sensitized in WTs (*p* < 0.001) but no change was observed in KOs (*p* = 0.97, Table 3, item b). The 5-min timebins of the intrasession data further show oxycodone-induced hyperlocomotion in WT but not KOs and sensitization of this response in only WTs over time (Fig. 2*B*, *p* < 0.01; Table 3, item c).

**Table 3:** Statistical analyses of the hyperlocomotor effects of oxycodone in MOR KO mice (**Fig. 2)**

Item	Figure	Experiment	Statistical test	Effect or Interaction	Main effect	WT	μ KO
**a**	2*A*	Oxycodone; total locomotion on day 1	Two-way ANOVA	Genotype, μ KO vs WT	*F*_(2,26)_ = 10.78, *p* < 0.0004	*n* = 7, day 1	*p* < 0.01 vs WT oxycodone, *n* = 9 *p* = 0.92 vs WT saline, *n* = 8
**b**	2*A*	Oxycodone sensitization; total locomotion	Two-way ANOVA	Genotype, μ KO vs WT	*F*_(2,26)_ = 5.6, *p* = 0.0095	*p* < 0.001 day 1 vs 3 *n* = 7	*p* = 0.97 day 1 vs 3, *n* = 9
**c**	2*B*	Oxycodone sensitization; intrasession analysis	Two-way ANOVA	Genotype, μ KO vs WT	*F*_(33,308)_ = 1.67, *p* = 0.015	*p* < 0.01, day 1 vs 3, *n* = 7	μ KO vs WT days 1 and 3 *p* < 0.001, *n* = 9

N.S.: not significant, N.A.: not applicable.

10.1523/ENEURO.0146-20.2020.f2-1 Extended Data Figure 2-1 *Oxycodone induced locomotion is absent in constitutive MOR KOs.* a. Oxycodone (10mg/kg s.c.) induces hyperlocomotion in WT mice (WT) that is absent in mice lacking all MORs (KO) on the first (**p<0.01 vs WT) and third (***p<0.001 vs WT) days of 3 consecutive days of oxycodone. There was no difference between the effect of oxycodone in KO mice with that of saline in WT mice (p=0.92). b. The intrasession data (shown here in 5’ timebins for the 60’ test) further show the lack of effect of oxycodone in KO mice on days 1 and 3 (p<0.0001 vs WT at all timepoints for both days). WTs demonstrated a sensitization of this locomotor response (p<0.01) from day 1 to day 3 that was absent in KOs. Refer to Table 3 for statistical analyses. All data are shown as mean ± SEM with the individual datapoints included. Download Extended Data Figure 2-1, EPS file.

**Figure 3.**
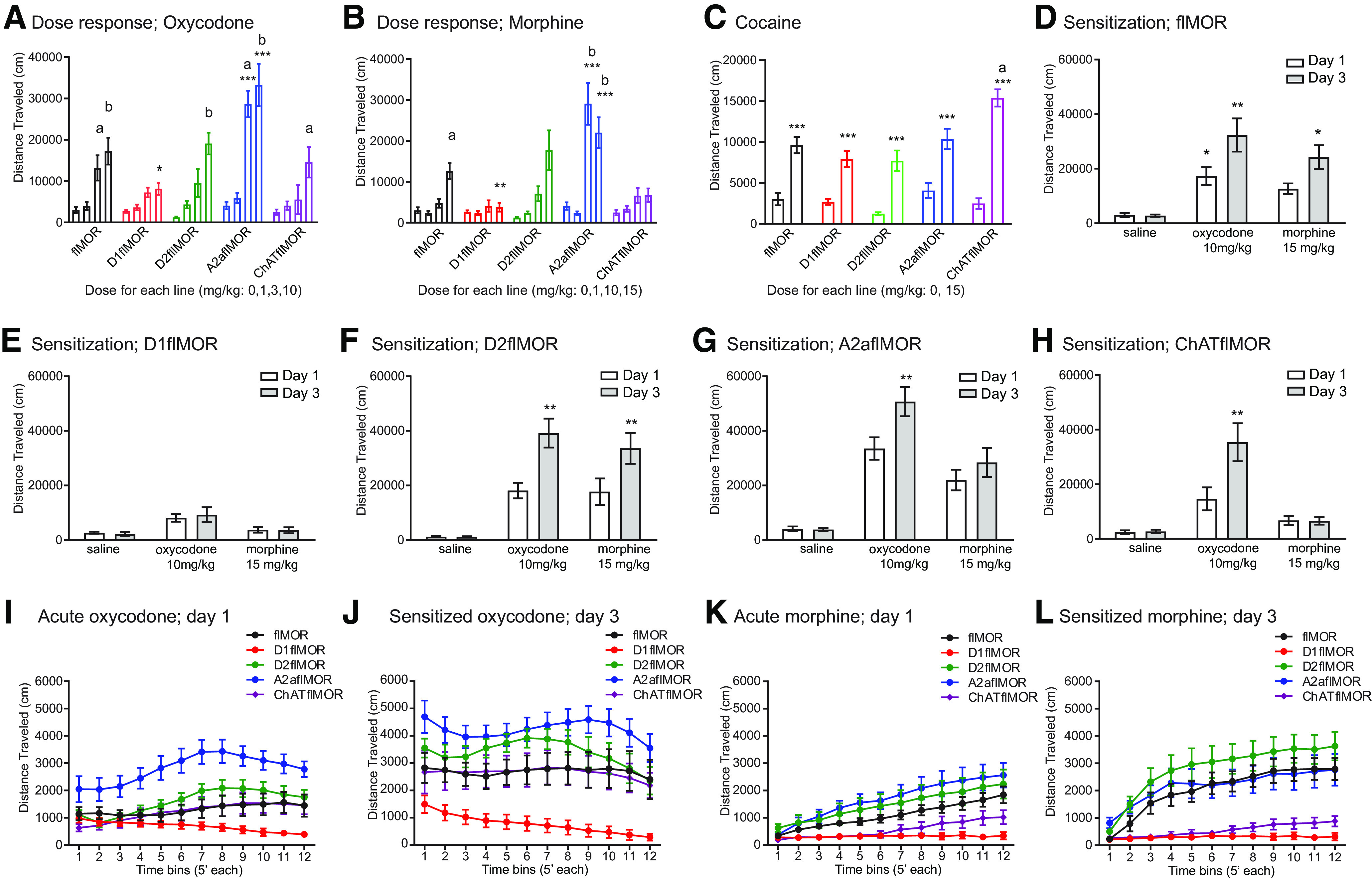
Selective MOR deletions define specific roles of D1 and A2a MOR populations in opioid-induced locomotion. ***A***, Oxycodone (0, 1, 3, 10 mg/kg) induced a dose-dependent increase in locomotion in flMORs, D2flMORs, A2aflMORs, and ChATflMORs, but not D1flMORs (a: *p* < 0.01 vs 0, b: *p* < 0.001 vs 0). In comparison with the control genotype, flMORs, D1flMORs showed a reduced effect of oxycodone at 10 mg/kg (*p < 0.05 vs flMOR of the same dose), whereas A2aflMORs showed an enhanced effect of oxycodone at 3 and 10 mg/kg (***p < 0.001 vs flMOR of the same dose). There was no effect of genotype following the vehicle (0) injection showing no effect of any of these deletions on basal locomotor activity. ***B***, Morphine (0, 1, 10, 15 mg/kg) also induced a dose-dependent increase in locomotor activity in flMORs, D2flMORs, and A2aflMORs but not in D1flMORs or ChATflMORs (a: *p* < 0.01 vs 0, b: *p* < 0.001 vs 0). Compared with control flMORs, this effect was enhanced in A2aflMORs (**p < 0.001 and ***p < 0.0001 vs flMOR of the same dose). ***C***, Cocaine (0, 15 mg/kg) induced hyperlocomotion in all lines when compared with saline (0; ***p ≤ 0.001), an effect that was enhanced in ChATflMORs (a: *p* < 0.001 vs flMORs). ***D–H***, Sensitization. After three consecutive days of repeated opioid injections, flMORs (***D***) and D2flMORs (***F***) showed an enhanced, or sensitized, response to both oxycodone and morphine. D1flMORs (***E***) did not show this enhanced effect to either opioid whereas A2aflMORs (***G***) and ChATflMORs (***H***) sensitized to oxycodone but not morphine (*p < 0.05 and **p < 0.01, respectively, vs day 1). ***I–L***, Intrasession locomotor analysis. This analysis assessed the locomotor response to oxycodone or morphine during each 60-min session on day 1 and day 3. ***I***, A single injection of oxycodone (10 mg/kg) on day 1 increased locomotor activity in D2flMORs (*p* < 0.05) and A2aflMORs (*p* < 0.0001), whereas flMORs, D1flMORs, and ChATflMORs showed no change in activity during the session. ***J***, After 3 d of repeated oxycodone administration, the locomotor activity of D1flMORs (*p* < 0.001) declined through the session and all other genotypes showed no change across time. ***K***, A single injection of morphine (15 mg/kg) on day 1 resulted in a within-session increase in locomotor activity in flMORs (*p* < 0.0001), D2flMORs (*p* < 0.0001), A2aflMORs (*p* < 0.0001), and ChATflMORs (*p* < 0.01), but not D1flMORs. ***L***, After 3 d of repeated morphine administration, a similar pattern emerged as on day 1 with flMORs (*p* < 0.0001), D2flMORs (*p* < 0.0001), A2aflMORs (*p* < 0.0001), and ChATflMORs (*p* < 0.05), but not D1flMORs, showing a within session increase in locomotor activity. Refer to Table 4 for statistical analyses. All data are shown as mean ± SEM, and individual datapoints are shown in Extended Data Figure 3-1 for which this legend also applies.

We then examined the dose–response relationship of oxycodone using 0 (saline), 1, 3, and 10 mg/kg subcutaneously in each of the genotypes (Fig. 3*A*). We found no effect of genotype following saline suggesting no effect of these deletions on basal locomotion (Table 4, item a). However, a significant dose by genotype interaction was found following oxycodone (*p* < 0.001, Table 4, item b). (1) Dose. When compared with the saline group of the same genotype, the 1 mg/kg dose of oxycodone had no effect, but 3 and 10 mg/kg of oxycodone induced hyperlocomotion in control flMORs (*p* = 0.007 and *p* = 0.0002, respectively), A2aflMORs (*p* < 0.0001 for both doses), and following 10 mg/kg in D2flMORs (*p* < 0.0001) and ChATflMORs (*p* = 0.0013). However, there was no effect of oxycodone in the D1flMORs (Table 4, item b). (2) Genotype. D1flMORs showed a decreased response compared with flMORs at 10 mg/kg (*p* = 0.04) whereas A2aflMORs showed a greater locomotor response than flMORs (*p* < 0.0001 for both doses). Neither ChATflMORs nor D2flMORs differed from flMORS (Table 4, item c).

**Table 4:** Statistical analyses of the hyperlocomotor effects of oxycodone, morphine, and cocaine in all lines (**Fig. 3)**

Item	Figure	Experiment	Statistical test	Effect or Interaction	Main effect	flMOR	D1flMOR	D2flMOR	A2aflMOR	ChATflMOR
**a**	3*A*	Oxycodone dose response: dose response (0 mg/kg or saline)	One-way ANOVA	Genotype	*F*_(4,36)_ = 2.54, *p* = 0.056	Reference genotype *n* = 8	N.S. *n* = 8	N.S. *n* = 8	N.S. *n* = 8	N.S. *n* = 9
**b**	3*A*	Oxycodone dose response: dose effect	Two-way ANOVA	Genotype × dose	*F*_(12,145)_ = 3.76, *p* < 0.001	3 mg; *p* = 0.007 vs 0, 10 mg; *p* = 0.0002 vs 0 *n* = 8–12	N.S. *n* = 8–11	3 mg; *p* = 0.56 vs 0, 10 mg; *p* < 0.0001 vs 0, *n* = 8	3 mg; *p* < 0.0001 vs 0, 10 mg; *p* < 0.0001 vs 0, *n* = 6–9	3 mg; *p* = 0.80 vs 0, 10 mg; *p* = 0.0013 vs 0 *n* = 5–9
**c**	3*A*	Oxycodone dose response: genotype effect	Two-way ANOVA	Genotype × dose	*F*_(12,145)_ = 3.76, *p* < 0.001	Reference genotype	10 mg: *p* = 0.04	N.S.	3 mg; *p* = 0.0001, 10 mg; *p* < 0.0001	N.S.
**d**	3*B*	Morphine dose response: dose effect	Two-way ANOVA	Genotype × dose	*F*_(12,148)_ = 5.7, *p* < 0.001	10 mg; N.S. 15 mg; *p* = 0.003 vs 0 *n* = 8–11	N.S. *n* = 8–11	10 mg; N.S. 15 mg; *p* < 0.0001 *n* = 8–11	10 mg; *p* < 0.0001, 15 mg; *p* < 0.0001 *n* = 5–9	N.S. *n* = 6–9
**e**	3*B*	Morphine dose response: genotype effect	Two-way ANOVA	Genotype × dose	*F*_(12,148)_ = 5.7, *p* < 0.001	Reference genotype	15 mg: *p* = 0.004	N.S.	10 mg; *p* = 0.0001, 15 mg; *p* = 0.004	N.S.
**f**	3*C*	Cocaine; dose effect	Two-way ANOVA	Treatment × genotype	*F*_(4,83)_ = 3.77, *p* = 0.0073	*p* < 0.0001 Cocaine, *n* = 21; saline *n* = 9	*p* = 0.0018 Cocaine, *n* = 9; saline *n* = 14	*p* = 0.0010 Cocaine, *n* = 9; saline *n* = 7	*p* = 0.0005 Cocaine, *n* = 9; saline *n* = 9	*p* < 0.0001 Cocaine, *n* = 10; saline *n* = 7
**g**	3*C*	Cocaine; genotype effect	Two-way ANOVA	Treatment × genotype	*F*_(4,83)_ = 3.77, *p* = 0.0073	Reference genotype	N.S. for saline and cocaine	N.S. for saline and cocaine	N.S. for saline and cocaine	Saline; N.S. Cocaine; *p* < 0.0005
**h**	3*D*	Locomotor sensitization; flMORs	Two-way ANOVA	Genotype × day	*F*_(2,27)_ = 3.9, *p* = 0.049	Oxycodone; *p* = 0.002 morphine; *p* = 0.018 *n* = 11 for both, saline *n* = 8				
**i**	3*E*	Locomotor sensitization; D1flMORs	Two-way ANOVA	Genotype × day	*F*_(2,25)_ = 0.6, *p* = 0.56		N.S. oxycodone *n* = 8, morphine *n* = 11, saline *n* = 8			
**j**	3*F*	Locomotor sensitization; D2flMORs	Two-way ANOVA	Genotype × day	*F*_(2,23)_ = 12.24, *p* = 0.0002			Oxycodone; *p* < 0.0001, *n* = 7, morphine; *p* < 0.0001 *n* = 11, saline *n* = 8		
**k**	3*G*	Locomotor sensitization; A2aflMORs	Two-way ANOVA	Genotype × day	*F*_(2,25)_ = 8.23, *p* = 0.0018				Oxycodone; *p* < 0.0001 *n* = 11, morphine; *p* = 0.15, *n* = 9, saline *n* = 8	
**l**	3*H*	Locomotor sensitization; ChATflMORs	Two-way ANOVA	Genotype × day	*F*_(2,23)_ = 11.53, *p* = 0.0003					Oxycodone; *p* < 0.0001, *n* = 8, morphine; N.S., *n* = 9, saline; *n* = 9
**m**	3*I*	Oxycodone intrasession analysis: Day 1	LMM	Genotype × timebin	χ^2^ = 11.882, *p* = 0.018	N.A.	N.S., *n* = 9	N.S., *n* = 8	N.S., *n* = 11	N.S., *n* = 9
**n**	3*J*	Oxycodone intrasession analysis: day 1	LMM	Timebin	χ^2^ = 31.215, *p* < 0.0001	N.S., *n* = 11	*p* = 0.076, *n* = 9	*p* = 0.016, *n* = 8	*p* < 0.0001, *n* = 11	N.S., *n* = 9
**o**	3*K*	Oxycodone intrasession analysis: day 3	LMM	Genotype × timebin	N.S.	N.A.	N.S., *n* = 9	N.S., *n* = 8	N.S., *n* = 11	N.S., *n* = 9
**p**	3*K*	Oxycodone intrasession analysis: day 3	LMM	Timebin	χ^2^ = 12.66, *p* = 0.027	N.S., *n* = 11	*p* < 0.001, *n* = 9	N.S., *n* = 8	N.S., *n* = 11	N.S., *n* = 9
**q**	3*L*	Morphine intrasession analysis: day 1	LMM	Genotype × timebin	χ^2^ = 21.239, *p* < 0.001	N.A.	*p* = 0.002	N.S.	N.S.	N.S.
**r**	3*L*	Morphine intrasession analysis: day 1	LMM	Timebin	χ^2^ = 54.796, *p* < 0.0001	*p* < 0.0001	N.S., *p* = 0.79	*p* < 0.0001	*p* < 0.0001	*p* = 0.002
**s**	3*M*	Morphine intrasession analysis: day 3	LMM	Genotype × timebin	χ^2^ = 32.962, *p* < 0.0001	N.A.	*p* < 0.0001	N.S.	N.S.	*p* < 0.0001
**t**	3*M*	Morphine intrasession analysis: day 3	LMM	Timebin	χ^2^ = 64.194, *p* < 0.0001	*p* < 0.0001	N.S., *p* = 0.56	*p* < 0.0001	*p* < 0.0001	*p* = 0.03

N.S.: not significant, N.A: not applicable.

10.1523/ENEURO.0146-20.2020.f3-1 Extended Data Figure 3-1 *Selective MOR deletions defines specific roles of D1 and A2a MOR populations in opioid-induced locomotion.* **a.** *Oxycodone* (0, 1, 3, 10mg/kg) induced a dose-dependent increase in locomotion in flMORs, D2flMORs, A2aflMORs and ChATflMORs, but not D1flMORs (a: p<0.01 vs 0, b: p<0.001 vs 0). In comparison with the control genotype, flMORs, D1flMORs showed a reduced effect of oxycodone at 10mg/kg (*p<0.05 vs flMOR of the same dose), whereas A2aflMORs showed an enhanced effect of oxycodone at 3 and 10mg/kg (***p<0.001 vs flMOR of the same dose). There was no effect of genotype following the vehicle (0) injection showing no effect of any of these deletions on basal locomotor activity. **b.** *Morphine* (0, 1, 10, 15 mg/kg) also induced a dose-dependent increase in locomotor activity in flMORs, D2flMORs and A2aflMORs but not in D1flMORs or ChATflMORs (a: p<0.01 vs 0, b: p<0.001 vs 0). Compared with control flMORs this effect was enhanced in A2aflMORs (**p<0.001 and ***p<0.0001 vs flMOR of the same dose). **c.** *Cocaine* (0, 15mg/kg) induced hyperlocomotion in all lines when compared with saline (0), (***; p< or =0.001), an effect that was enhanced in ChATflMORs (a: p<0.001 vs flMORs). **d-h.** *Sensitization.* After 3 consecutive days of repeated opioid injections, flMORs (**d)**, and D2flMORs (**f**) showed an enhanced, or sensitized, response to both oxycodone and morphine. D1flMORs (**e**) did not show this enhanced effect to either opioid whereas A2aflMORs (**g**) and ChATflMORs (**h**) sensitized to oxycodone but not morphine (*,** p<0.05 and 0.01 respectively vs day 1). **i-l** *Intrasession locomotor analysis*. This analysis assessed the locomotor response to oxycodone or morphine during each 60 min session on day 1 and day 3 **i**. A single injection of oxycodone (10 mg/kg) on day 1 increased locomotor activity in D2flMORs (p<0.05) and A2aflMORs (p<0.0001) whereas flMORs, D1flMORs and ChATflMORs showed no change in activity during the session. **j.** After 3 days of repeated oxycodone administration, the locomotor activity of D1flMORs (p<0.001) declined through the session and all other genotypes showed no change across time. **k.** A single injection of morphine (15 mg/kg) on day 1 resulted in a within-session increase in locomotor activity in flMORs (p<0.0001), D2flMORs (p<0.0001), A2aflMORs (p<0.0001) and ChATflMORs (p<0.01), but not D1flMORs. **l**. After 3 days of repeated morphine administration, a similar pattern emerged as on day 1 with flMORs (p<0.0001), D2flMORs (p<0.0001), A2aflMORs (p<0.0001) and ChATflMORs (p<0.05), but not D1flMORs, showing a within session increase in locomotor activity. Refer to Table 4 for statistical analyses. All data are shown as mean ± SEM with the individual datapoints included. Download Extended Data Figure 3-1, EPS file.

#### Morphine

Our first experiments examined the dose-dependent locomotor effects of morphine using 0 (saline), 3, 10, and 15 mg/kg (subcutaneously; Fig. 3*B*). We observed a dose × genotype interaction (*p* < 0.001, Table 4, item d) as follows. (1) Dose. When compared with the group receiving saline of the same genotype, we found that 15 mg/kg morphine, but not any lower doses, induced hyperlocomotion in flMORs (*p* = 0.003) and D2flMORs (*p* < 0.0001). D1flMORs and ChATflMORs showed no response at any dose (Table 4, item d) whereas A2aflMORs showed hyperlocomotion after both 10 and 15 mg/kg (*p* < 0.0001 for both doses), but not 3 mg/kg. (2) Genotype. Between genotype analysis (Table 4, item e) showed a similar effect of genotype following morphine as oxycodone treatment in that, when compared with flMORs, A2aflMORs showed an enhanced response at the higher doses used, 10 (*p* = 0.0001) and 15 (*p* = 0.004) mg/kg, whereas D1flMORs showed a reduced response at 15 mg/kg (*p* = 0.004), but not 10 mg/kg. Both D2- and ChAT-flMORs were not different from flMORs.

#### Cocaine

To assess whether the changes in opioid-induced locomotor responses were generalizable to other drug classes, we determined the effect of genotype on cocaine-induced locomotion (15 mg/kg, s.c.; Fig. 3*C*). We found cocaine-induced locomotion in all genotypes (Fig. 3*C*, *p* ≤ 0.001; Table 4, item f) but this effect was enhanced in ChATflMORs (*p* < 0.0005, Table 4, item g).

### Locomotor sensitization

Repeated opioid exposure is well known to induce a sensitization of the initial hyperlocomotor response (Tao et al., 2017). This occurs concurrently with an increase in the incentive motivational properties of a drug and has been considered as a window into this property of drug-seeking behavior (Robinson and Berridge, 1993). To assess the role of each of these MOR populations in this phenomenon, we examined sensitization to oxycodone (10 mg/kg, s.c.), morphine (15 mg/kg, s.c.), and saline, over three consecutive days of drug exposure in all genotypes. The data were analyzed by two-way ANOVA to assess the effect of day and drug on the first and last days of the test. The flMORs showed a genotype × day interaction as both oxycodone (*p* = 0.002) and morphine (*p* = 0.02), but not saline, induced sensitization (Fig. 3*D*; Table 4, item h). The D1flMORs showed no sensitization effect following oxycodone or morphine and this response was not different from saline (Fig. 3*E*; Table 4, item i). The D2flMORs were similar to flMORs as they sensitized to both oxycodone (*p* < 0.0001) and morphine (*p* < 0.0001) but not saline (Fig. 3*F*; Table 4, item j). The A2aflMORs sensitized to oxycodone (*p* < 0.0001) but not to morphine or saline. (Fig. 3*G*; Table 4, item k). The ChATflMORs similarly sensitized to oxycodone (*p* < 0.0001), but not morphine or saline (Fig. 3*H*; Table 4, item l).

### Intrasession locomotor activity

We then defined the locomotion profile induced by each drug with each session using linear mixed model analysis to assess the effect of time and genotype. This was done using 5-min timebins on day 1 and day 3 of 10 mg/kg oxycodone or 15 mg/kg morphine. (1) There was a genotype × time interaction on day 1 of oxycodone (Fig. 3*I*, *p* < 0.0001; Table 4, item m). The D2flMORs (Table 4, item n, *p* = 0.02) and A2aflMORs (*p* < 0.0001), but not flMORs, D1- or ChAT-flMORs showed a change in locomotor activity within the session. (2) We did not find a timebin × genotype interaction (Table 4, item o) on day 3 of oxycodone. However, the D1flMORs showed decreased activity over time (Fig. 3*J*, *p* < 0.001; Table 4, item p), but no other change in activity over time was observed in other lines. (3) There was a genotype × time interaction on day 1 of morphine (Fig. 3*K*, *p* < 0.0001; Table 4, item q), with D1flMORs showing a different locomotor profile than flMORs (*p* = 0.002). Further *post hoc* analyses showed that flMORs (Table 4, item r, *p* < 0.0001), D2flMORs (*p* < 0.0001), A2aflMORs (*p* < 0.0001), and ChATflMORs (*p* = 0.002), but not D1flMORs, increased their locomotor activity during the session. (4) We also observed a significant genotype × time interaction on day 3 (Fig. 3*L*, *p* < 0.0001; Table 4, item s), with both D1flMOR (*p* < 0.0001) and ChATflMOR (*p* < 0.001) showing less activity during the session than the flMORs. Further *post hoc* analyses showed that flMORs (Table 4, item t, *p* < 0.0001), D2flMORs (*p* < 0.0001), A2aflMORs (*p* < 0.0001), and ChATflMORs (*p* = 0.03), but not D1flMORs, increased their locomotor activity during this session.

### Selective MOR deletions define specific roles of A2a and ChAT MOR populations in opioid IVSA

Although opioid-induced locomotion and sensitization of this response have been used as an index of reward behaviors (Robinson and Berridge, 1993; Stewart and Badiani, 1993), IVSA is considered as a more direct measure of reward seeking and addiction (Everitt et al., 2018). We therefore examined whether deleting MORs from these neurons altered opioid IVSA through an indwelling jugular catheter under a short-access FR1 schedule. Each of the phases of the IVSA protocol (remifentanil acquisition, oxycodone maintenance and extinction) were analyzed separately and results presented for each of the following four parameters; active and inactive lever presses, reinforcers earned and lever choice as shown by the percent of active lever/total lever presses made.

### Remifentanil acquisition

**Figure 4.**
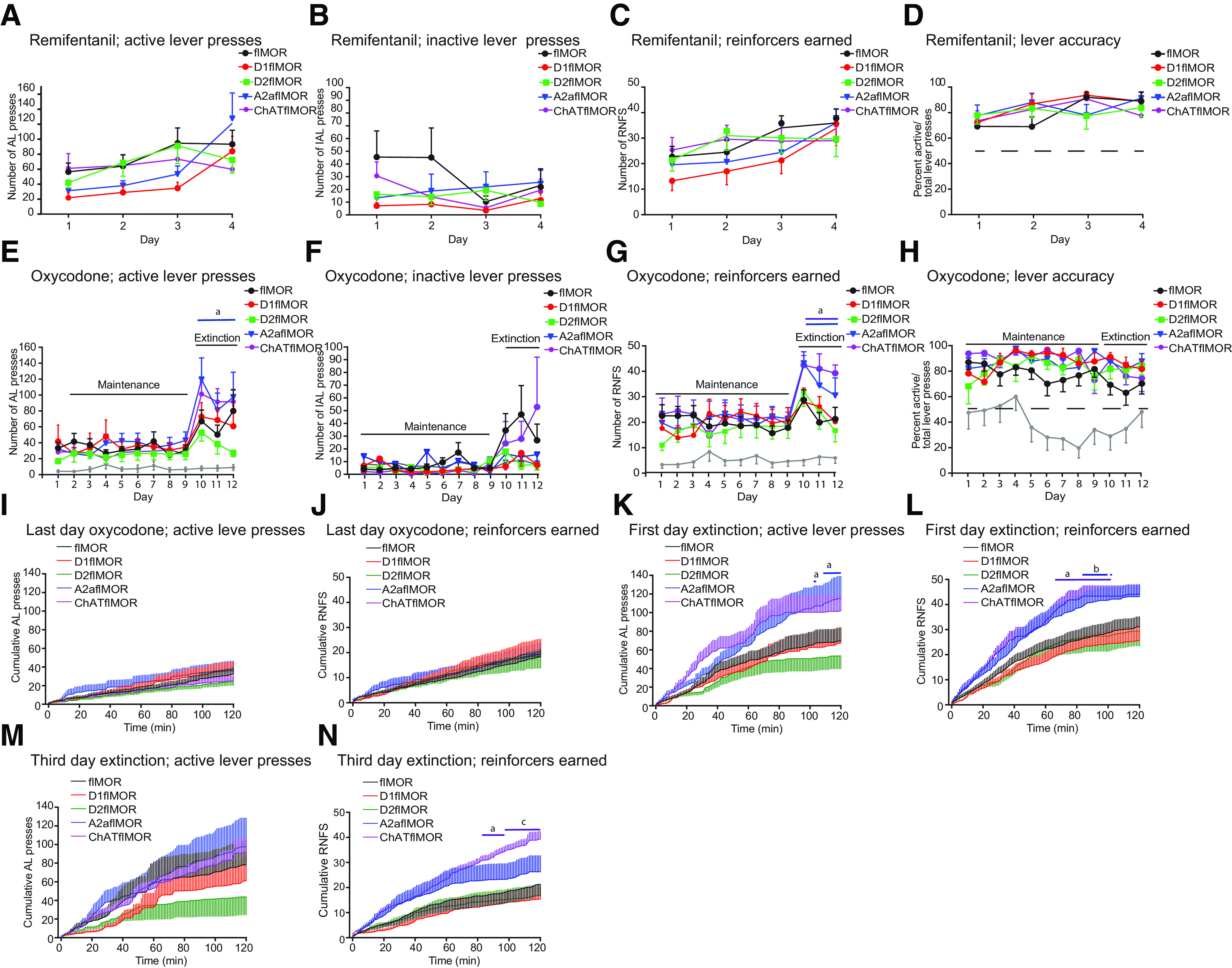
Selective MOR deletions define specific roles of A2a and ChAT populations in an opioid self-administration profile**. *A–D***, Acquisition. During this short acquisition phase (days 1–4) during which remifentanil was self-administered there was no effect of genotype and no interaction or a main effect of genotype on any of the four parameters measured; active lever presses (***A***), inactive lever presses (***B***), reinforcers earned (***C***), or the percent active lever presses made (***D***). ***E–H***, Maintenance and extinction. Mice were then transitioned to oxycodone self-administration for 9 d followed by 3 d of extinction. When compared with mice self-administering saline, those that self-administered oxycodone made more active lever presses (*p* < 0.05), earned more reinforcers (*p* < 0.05), and showed a preference for the active over inactive lever (*p* < 0.0001) during the maintenance and extinction session. ***E***, During the extinction but not maintenance phases, A2aflMORs made more active lever presses than flMORs (*p* < 0.05). ***F***, There was no effect of genotype on the number of inactive lever presses at any stage. ***G***, Similar to the number of active lever presses made, A2aflMORs and ChATflMORs earned more reinforcers than flMORs during extinction (a: *p* < 0.05)**. *H***, There was no effect of genotype on active lever preference as shown by the percent active lever/total lever presses. ***I–N***, Within session analysis of the cumulative number of active lever presses made and reinforcers earned during the 2-h session was assessed on three specific days; the last day of oxycodone (day 9) and the first (day 10) and third (day 12) days of extinction. This shows no effect of genotype on the last day of oxycodone for either the cumulative active lever presses (***I***) or reinforcers (***J***) earned. ***K***, However, on the first day of extinction, A2aflMORs made more active lever presses than flMORs (a: *p* < 0.05 vs flMOR at 103 and 104 and 110–120 min). ***I***, A similar effect was seen in the reinforcers earned during this session when A2aflMORs earned more reinforcers (a: *p* < 0.05 vs flMOR at 87–99 and 103 min) as did ChATflMORs (b: *p* < 0.05 vs flMOR at 69–102 min). ***N***, On the third day of extinction, there was no further effect of genotype on the number of active lever presses made. ***M***, However, the ChATflMORs showed an increase in reinforcers earned on the third day of extinction (a and c: *p* < 0.05 and *p* < 0.01, respectively, vs flMOR at 82–120 min). Refer to Table 5 for statistical analyses. All data are shown as mean ± SEM.

Remifentanil, a fast-acting opioid, was used to establish the association of an active lever press with an opioid infusion and associated cues. During this short acquisition phase, we did not find a genotype × day interaction or any main effect of genotype on any of the four parameters measured; (Fig. 4*A–D*, respectively). However, we found a main effect of day on active lever presses made (*p* < 0.0001, χ^2^ = 21.017; Table 5, item a), reinforcers earned (*p* < 0.0001, χ^2^ = 19.132; Table 5, item b), and percentage active lever presses (*p* < 0.01, χ^2^ = 9.730; Table 5, item c), but not inactive lever presses, showing that all lines acquired this self-administration behavior but there was no effect of genotype.

**Table 5:** Statistical analyses of the IVSA profile in all lines (**Fig. 4)**

Item	Figure	Experiment	Statistical test	Effect or interaction	Main effect	flMOR	D1flMOR	D2flMOR	A2aflMOR	ChATflMOR
**a**	3*A*	Remifentanil acquisition	LMM	AL day effect	*p* < 0.0001, χ^2^ = 21.017	*n* = 15	*n* = 12	*n* = 10	*n* = 12	*n* = 12
**b**	3*C*	Remifentanil acquisition	LMM	RNFS earned day effect	*p* < 0.0001, χ^2^ = 19.132					
**c**	3*D*	Remifentanil acquisition	LMM	Percent AL presses day effect	*p* < 0.01, χ^2^ = 9.730					
**d**	3*E*	Oxycodone maintenance	LMM	AL presses *×* Treatment Oxycodone vs. Saline	*p* < 0.001, χ^2^ = 10.926	*p* < 0.001, χ^2^ = 11.601, *n* = 14	*p* < 0.01, χ^2^ = 6.764, *n* = 9	*p* < 0.001, χ^2^ = 12.806, *n* = 9	*p* < 0.01, χ^2^ = 10.545, *n* = 8	*p* < 0.001, χ^2^ = 13.68, *n* = 7
**e**	3*G*	Oxycodone maintenance	LMM	RNFS × treatment Oxycodone vs. Saline	*p* < 0.0001, χ^2^ = 16.051	*p* < 0.001, χ^2^ = 11.435	*p* < 0.01, χ^2^ = 8.434	*p* < 0.01, χ^2^ = 9.339	*p* < 0.001, χ^2^ = 10.981	*p* < 0.001, χ^2^ = 14.648
**f**	3*G*	Oxycodone maintenance	LMM	Percent AL presses treatment × day oxycodone vs saline	*p* < 0.0001, χ^2^ = 34.81	*p* < 0.0001, χ^2^ = 15.957	*p* < 0.0001, χ^2^ = 20.334	*p* < 0.01, χ^2^ = 7.918	*p* < 0.0001, χ^2^ = 16.952	*p* < 0.0001, χ^2^ = 19.941
**g**	3*E*	Extinction; transition	LMM	AL presses treatment × day oxycodone vs. saline	*p* < 0.01, χ^2^ = 8.296					
**h**	3*G*	Extinction; transition	LMM	RNFS treatment × day oxycodone vs. saline	*p* < 0.01, χ^2^ = 7.047					
**i**	3*E*	Extinction; transition	LMM	AL presses day effect within oxycodone treated	*p* < 0.0001, χ^2^ = 29.255					
**j**	3*F*	Extinction; transition	LMM	IAL presses day effect within oxycodone treated	*p* < 0.01, χ^2^ = 9.396					
**k**	3*G*	Extinction; transition	LMM	RNFS day effect within oxycodone treated	*p* < 0.0001, χ^2^ = 25.725					
**l**	3*G*	Extinction	LMM	AL presses × genotype within oxycodone treated	*p* = 0.050, χ^2^ = 9.480	N.A.	N.S.	N.S.	*p* = 0.043	N.S.
**m**	3*G*	Extinction	LMM	IAL presses × genotype within oxycodone treated	*p* = 0.057, χ^2^ = 9.148	N.A.	N.S.	N.S.	*p* = 0.025	N.S.
**n**	3*G*	Extinction	LMM	RNFS earned × genotype within oxycodone treated	*p* = 0.018, χ^2^ = 11.934	N.A.	N.S.	N.S.	*p* = 0.031	*p* = 0.007
**o**	3*G*	Extinction	LMM	RNFS earned day effect	*p* < 0.001, χ^2^ = 12.979	*p* < 0.001, *t*_(50)_ = –4.124	*p* < 0.001, *t*_(53)_ = –3.964	*p* < 0.001, *t*_(55)_ = –3.946	*p* < 0.001, *t*_(55)_ = –3.135	*p* < 0.001, *t*_(56)_ = –2.726
**p**	3*I*	Last day AL oxycodone	Two-way ANOVA	Genotype × time	*F*_(480,5160)_ = 0.45, *p* > 0.99					
**q**	3*L*	Last day oxycodone RNFS	Two-way ANOVA	Genotype × time	*F*_(480,4920)_ = 0.2, *p* > 0.99					
**r**	3*J*	First day extinction AL	Two-way ANOVA	Genotype × time	*F*_(480,4920)_ = 1.44, *p* < 0.0001	Reference genotype	N.S.	N.S.	*p* < 0.05	N.S.
**s**	3*M*	First day extinction RNFS	Two-way ANOVA	Genotype × time	*F*_(480,4800)_ = 1.66, *p* < 0.0001	Reference genotype	N.S.	N.S.	*p* < 0.05	*p* < 0.05
**t**	3*J*	Third day extinction AL	Two-way ANOVA	Genotype × time	*F*_(480,5160)_ = 0.42, *p* > 0.99	Reference genotype	N.S.	N.S.	N.S.	N.S.
**u**	3*M*	Third day extinction RNFS	Two-way ANOVA	Genotype × time	*F*_(480,5280)_ = 2.02, *p* < 0.0001	Reference genotype	N.S.	N.S.	N.S.	*p* < 0.05 and *p* < 0.01

N.S.: not significant, N.A: not applicable.

### Oxycodone maintenance

The mice were then transitioned to oxycodone self-administration for 9 d. Compared with those on saline, mice receiving oxycodone made more active lever presses (Fig. 4*E*, *p* < 0.001; Table 5, item d), earned more reinforcers (Fig. 3*G*, *p* < 0.0001; Table 5, item e), and had a higher percentage active lever presses (Fig. 4*H*, *p* < 0.0001; Table 5, item f). There was no difference in the inactive lever presses made between the saline and oxycodone groups (Fig. 4*F*). There was no effect of genotype on any parameter.

### Extinction

Extinction has been shown to increase drug-seeking behavior following oxycodone self-administration (Hakimian et al., 2019). We similarly found that, when compared with saline, all genotypes showed a treatment × day interaction in the number of active lever presses made (*p* < 0.01; Fig. 4*E*; Table 5, item g) and reinforcers earned (*p* < 0.01; Fig. 4*G*; Table 5, item h), but not inactive lever presses (Fig. 4*F*) or percentage active lever presses (Fig. 4*D*) between the last day of oxycodone maintenance and the first day of extinction. *Post hoc* analyses showed an effect of oxycodone in that mice receiving oxycodone made more active lever presses (*p* < 0.0001; Table 5, item i), inactive lever presses (*p* < 0.0001, Table 5, item j) and earned more reinforcers (*p* < 0.0001; Table 5, item k) on the first day of extinction versus the last day of maintenance. No such transition effect was observed across any parameter in the saline group.

We then assessed the change in drug-seeking behavior over the 3 d of extinction in mice that had received oxycodone using LMM analysis. We found no genotype × day interaction, however there was a main effect of genotype on reinforcers earned (Fig. 4*G*, *p* < 0.05; Table 5, item n) with ChATflMORs (*p* < 0.01) and A2aflMORs (*p* < 0.05) earning more reinforcers than flMOR mice over these 3 d. There was a trend toward a main effect of genotype for active lever presses (Fig. 3*E*, *p* = 0.0507; Table 5, item l) and percentage active lever presses (Fig. 4*H*, *p* = 0.0571; Table 5, item m), with A2aflMORs showing increased active lever presses (*p* < 0.05) and percentage active lever presses (*p* < 0.05) made over these 3 d than the flMORs. We also observed a main effect of day on reinforcers earned (Fig. 4*G*, *p* < 0.0001; Table 5, item o) with all mice showing a decrease in reinforcers earned over the 3 d of extinction with no effect of genotype. No other effects were found for active lever presses, inactive lever presses and percentage active lever presses across these 3 d.

### Intrasession analysis

We also analyzed the cumulative frequency of active lever presses and reinforcers earned during the 2-h test on three specific days of the IVSA protocol (Fig. 4*I–N*). The first of these days, day 9 of the maintenance phase and the last day of oxycodone self-administration, showed a lack of genotype effect on either the cumulative active lever presses (Fig. 4*I*; Table 5, item p) or reinforcers earned (Fig. 4*J*; Table 5, item q). However, on the next day assessed, extinction day 1, A2aflMORS showed an increase in cumulative active lever presses (Fig. 4*K*, *p* < 0.05; Table 5, item r) and reinforcers earned (Fig. 4*L*, *p* < 0.05; Table 5, item s). ChATflMORs also earned more reinforcers than flMORs on this day (Fig. 4*L*, *p* < 0.05; Table 5, item s). By the third day of extinction, there was no effect of genotype on cumulative active lever presses (Fig. 4*M*; Table 5, item t), but there was an effect of genotype on cumulative reinforcers earned with ChATflMORs earning more reinforcers than flMORs during the last 40 min of the test (Fig. 4*N*; Table 5, item u).

## Discussion

**Figure 5.**
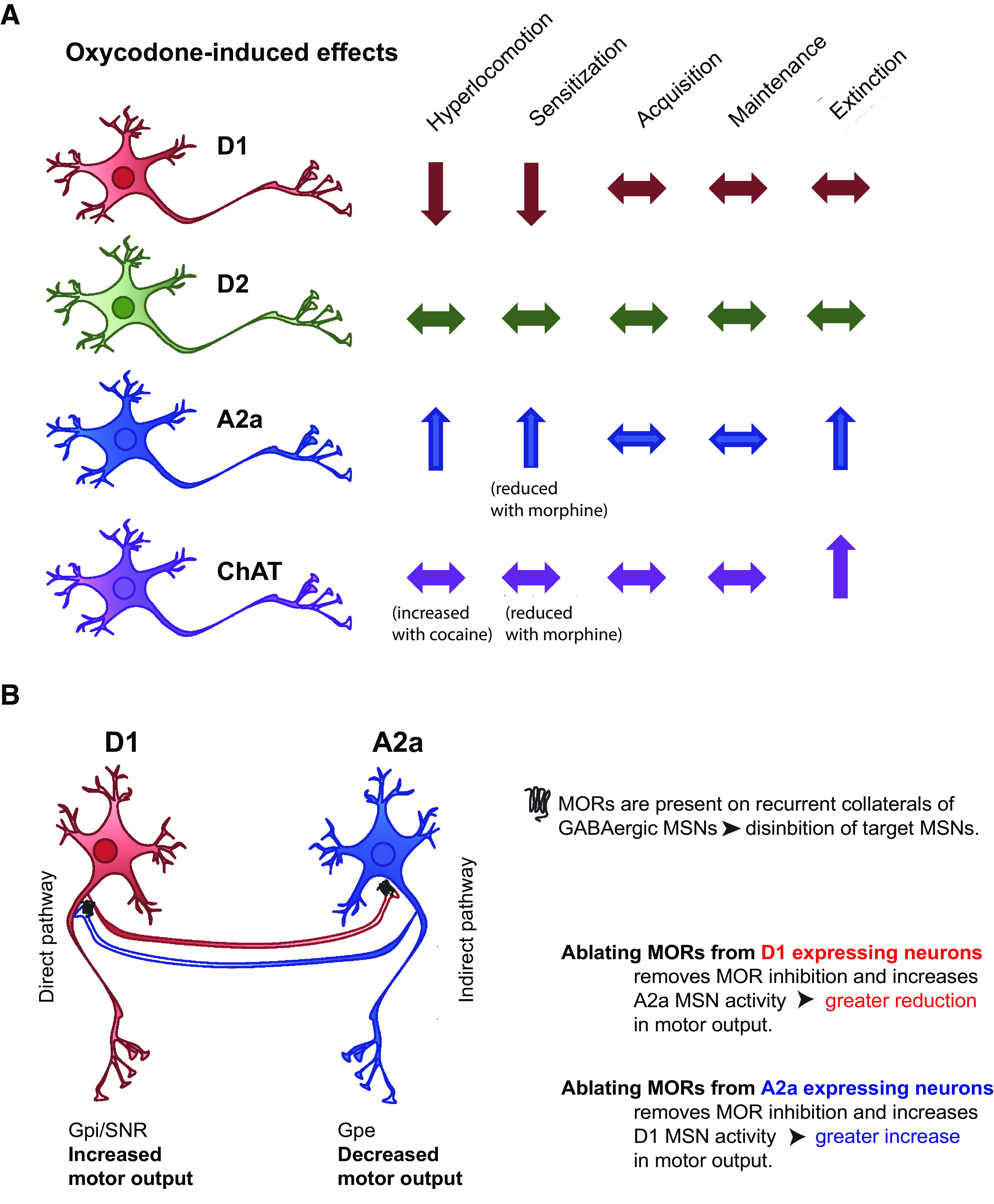
***A***, Summary of our findings. Deleting MORs from D1 neurons reduces oxycodone-induced hyperlocomotion and sensitization but does not alter the IVSA profile. Deleting MORs from D2 neurons alters neither the locomotor effects of oxycodone nor the IVSA profile whereas deleting MORs from A2a neurons increases oxycodone-induced hyperlocomotion and sensitization and also drug-seeking behaviors following opioid IVSA. Deleting MORs from ChAT neurons does not alter oxycodone-induced hyperlocomotion and sensitization but does increase the locomotor effect of cocaine and drug-seeking behaviors following opioid IVSA. ***B***, A possible mechanism by which MORs on D1 or A2a neurons alter striatal-mediated motor output. Removing MORs from D1 medium spiny neurons and so D1-A2a recurrent collateral increases A2a neuronal activity to reduce striatal motor output. Conversely removing MORs from A2a medium spiny neurons and so A2a-D1 recurrent collaterals increases D1 neuronal activity to increase striatal motor output.

These findings outline distinct roles for MORs on neuronal populations in behaviors associated with opioid-induced locomotion and reward behaviors. These are that selective ablation of MORs from D1 receptor-expressing neurons prevents opioid-induced locomotor hyperactivity as well as locomotor sensitization but has no effect on opioid IVSA. Second, removal of MORs from A2a neurons enhances opioid-induced hyperlocomotion, locomotor sensitization and drug-seeking behaviors during extinction following opioid IVSA. Third, ablation of MORs from ChAT neurons results in an agonist-dependent hyperlocomotor effect whereby morphine fails to elicit dose-dependent locomotor hyperactivity or sensitization yet oxycodone-induced effects are similar to control flMOR mice. These mice also show an increase in drug-seeking behavior during extinction. Fourth, despite the common theory that A2a receptor expression is equivalent to D2 receptor expression in medium spiny neurons, our data suggests that the A2a cre deletes MORs from only a subset of D2 medium spiny neurons and, that, in stark contrast to MOR deletion from A2a neurons, MOR deletion from D2 neurons results in no discernible change in these reward-based behaviors (Fig. 5*A*).

Our study shows that MORs on D1 neurons are required for the initial locomotor and sensitization response to morphine and oxycodone. The effect of morphine is in line with a previous study in which the expression of MORs in only D1 neurons in striatal patches in an otherwise null background reinstated morphine-induced locomotion (Cui et al., 2014). Together these 2 findings demonstrate both the requirement and necessity of this MOR population for this striatal-mediated output. This may be a result of MORs on D1 recurrent collaterals inhibiting D2 neurons to reduce striatal output and attenuate the motor effect of opioids, as modeled in Figure 5*B*. Another possibility is that these receptors are required for the release of dopamine in the VTA (Cui et al., 2014), which is required for this response (Steidl et al., 2017). In regards our IVSA findings, the lack of effect of the D1 MOR deletion in the acquisition of oxycodone IVSA is in contrast with previous work (Cui et al., 2014), perhaps as other MOR populations such as those within the matrix, are also involved in the acquisition phase of this behavior. It is also possible that this is an example of an opioid-specific effect in which the faster-acting opioid, remifentanil, used in (Cui et al., 2014), results in greater lever pressing behavior than oxycodone.

As D2 receptors are expressed on cholinergic interneurons (Weiner et al., 1991), the A2a cre line has been used to selectively target D2 medium spiny neurons (Fink et al., 1992; Rosin et al., 2003; Wang et al., 2019). Our findings show that this A2a-MOR population is an apparent subset of D2 medium spiny neurons that controls the locomotor sensitivity to oxycodone and morphine and drug-seeking behavior during extinction. These inhibitory receptors may be on some D2-D1 collaterals (Taverna et al., 2008), where their deletion allows an earlier threshold to be reached to increase striatal motor output, as modeled in Figure 5*B*. As MORs on cholinergic interneurons remains intact and, surprisingly, MORs are also present on some D2 striatal neurons, their deletion displays a remarkably different and striking phenotype from D2flMORs. This could reflect a role of this striatal population or an extrastriatal neuronal population that expresses both A2a and μ opioid but not necessarily D2 receptors. As regards MORs on D2+ neurons, we find that these receptors influence neither opioid-induced locomotion nor opioid IVSA.

While deleting MORS from D1, D2 and A2a neurons was performed to identify their role in GABAergic striatal neurons, deleting MORs from cholinergic interneurons examines the role of these receptors in altering cholinergic neuronal activity. These neurons form 1–3% of the striatal population yet they are remarkably influential in controlling striatal circuits (Gritton et al., 2019) and output, and both MORs and δ-opioid receptors strongly inhibit their rhythmic activity to affect behavior (Bertran-Gonzalez et al., 2013; Ponterio et al., 2013). Activation of MORs could affect glutamate or acetylcholine release and subsequent dopamine release from nearby terminals (Yorgason et al., 2017) to alter the activity of local circuits (for review, see Clarke and Adermark, 2015; Berke, 2018). Omission of an expected reward induces a dip in dopamine release, a negative reward prediction error (RPE) accompanied by a pause in cholinergic interneuron activity (Hart et al., 2014) to affect local D1 and D2 medium spiny neuron activity (Mamaligas and Ford, 2016). Deleting MORs from these neurons may prevent the encoding of an RPE and facilitate drug-seeking, as shown by an increase in cue-induced reinforcers earned, but not active lever presses, during extinction (Fig. 4*M*,*N*).

The rapid increase in hyperlocomotion following oxycodone and the sustained, gradual increase in hyperlocomotion following morphine (Fig. 3*I–L*) is likely because of the different plasma-kinetic (PK) profiles of these two drugs. Oxycodone has a higher percentage of unbound drug in the blood and a 100-fold greater influx rate than morphine (Boström et al., 2008). This results in a 6-fold higher ratio of unbound oxycodone in the brain: blood and a higher unbound steady state in the brain (Boström et al., 2006, 2008) likely explaining the larger increase in dopamine release following intravenous oxycodone than intravenous morphine (Vander Weele et al., 2014). The ligand-dependent and genotype-dependent effect of morphine but not oxycodone in ChATflMORs further suggests that this receptor population is more sensitive to the PK profile of each ligand. This could be because of a time-dependent effect of these receptors in modulating intrinsic cholinergic interneuron activity and the control of local circuitry.

There are several limitations of this study. One is that we have used the loxP-Cre recombinase system to achieve developmental deletion of MORs from various neuronal populations (Gong et al., 2007). For the most part these populations are striatal where the co-expression of MORs with D1 or D2 receptors can be used to define different medium spiny neuron populations (Gerfen et al., 1990; Weiner et al., 1991). However, dopamine neurons project to various brain regions in addition to the striatum, the hippocampus, amygdala, and prefrontal cortex. The behavioral outcomes in this study may therefore be influenced by MOR expression on dopamine circuits outside the striatum. For example, MOR expression on the intercalated neurons of the amygdala (Gregoriou et al., 2019), and in the globus pallidus (Weiner et al., 1991; Delfs et al., 1994) may influence these reward-related behaviors. MOR expression on cholinergic neurons of the medial habenula (Gardon et al., 2014) may also influence reward behaviors (Boulos et al., 2020) and MORs and ChAT co-expression in secretomotor neurons of the colon suggests gut function may be altered in ChATflMORs (Galligan and Akbarali, 2014). Further studies could also assess the role of MORs in different striatal subregions such as in patches or matrix, dorsal ventral striatum and co-expression with both D1 and D2 receptors (Soares-Cunha et al., 2016). An additional limitation is that we did not assess the effect of the cre insertion alone as this would have required further back-crossing of all lines.

Striatal D1 and D2 neurons are traditionally considered to have opposing effects on striatal motor patterns resulting in a coordinated motor activity. In this simple model, activating D1 neurons of the direct pathway increases striatal output to facilitate movement whereas activating D2 neurons of the indirect pathway inhibits competing motor patterns and inhibits movement (Kravitz et al., 2010). This model has been expanded and developed to include several interacting factors that influence the threshold of these outputs by recurrent collaterals between D1 and D2 neurons (Bahuguna et al., 2015), regulation by different interneurons (Taverna et al., 2008), and the regional and compartmental expression patterns of D1 and D2 (Cui et al., 2014; Oude Ophuis et al., 2014). Nevertheless, the opposing and complimentary effects of medium spiny neuron activation remains a central component of their activity. We show that the effect of deleting MORs from D1 and A2a neurons resembles such complementation, albeit the inverse, as it is the absence of MORs from D1 or A2a neurons that reduces or facilitates motor output, respectively. We propose that this can be explained by the presence of these G*_io_*-coupled receptors on recurrent medium spiny neuron collaterals, as shown by the schematic model in Figure 5*B*. The roles of D1 and D2 medium spiny neurons in mediating reward are also seen as divergent yet complementary in that D1 neurons mediate drug reinforcement and positive reward behaviors, whereas the D2s mediate aversion or ambivalence and are active during withdrawal (Koo et al., 2014; Cole et al., 2018). In addition, D1 and D2 receptors also play complementary but opposing roles in learning value-based and motivated behaviors, an important component of the change in reward value during extinction (Verharen et al., 2019). In regards the roles of MORs on these neurons, we show that rather than mediating positive reinforcement during the initial stages of opioid reward, that it is MORs on A2a or ChAT neurons that are important in controlling drug seeking during extinction, a period of increased anxiety and negative affect (Carmack et al., 2019). Additional studies to further define the effect of these deletions on A2a or ChAT neurons under different physiological conditions such as an increase in stress following periods of abstinence, or chronic pain, are needed to enhance our understanding of the complex and interrelated roles of these MOR populations.

## Synthesis

Reviewing Editor: Karen Szumlinski, University of California at Santa Barbara Decisions are customarily a result of the Reviewing Editor and the peer reviewers coming together and discussing their recommendations until a consensus is reached. When revisions are invited, a fact-based synthesis statement explaining their decision and outlining what is needed to prepare a revision will be listed below. The following reviewer(s) agreed to reveal their identity: Camron Bryant, Olivier George.

Upon consultation with two experts with expertise in the behavioral genetics and neuropharmacology of the opioid system, we have reached the decision of "Revise and Re-review". This decision was based on the consensus that this report has the potential to be very impactful given the nature of the research approach (first study characterizing the effects of cell-specific targeting of MORs) of relevance not only to substance use disorders, but also pain processing. The report summarizes an impressive amount of work. That being said, this report could benefit from some major revision, but the majority of issues raised should be readily addressable within a 2-month time-frame. Both reviewers had issue with the lack of information in the figure presentation. In addition to this common concern, each reviewer has provided a very detailed list of independent issues/concerns that we would like to see addressed in a revised manuscript. We understand with the Covid-19 shut-down that more than 2 months‘ time may be required to complete the revision. If that turns out to be the case, please contact eNeuro and the Editorial staff we will work hard to accommodate any such request.

Reviewer 1:

Comment on Statistics: Yes, in part, but Sex is never mentioned as a biological factor that could be included in the statistical model. They tested both females and males but from what I could tell, there is no mention of how many females vs. males were used per genotype per treatment per experiment. We know there are robust differences in opioid-induced locomotor effects (females > males, generally) so if there is any imbalance in the sample sizes, this could confound the results.

No power analysis is provided to give a rationale for the sample sizes employed.

ummary

• Rationale: Lack of knowledge regarding cell types in striatum that contribute to opioid-induced locomotor stimulation and reinforcement. Here, they targeted 4 striatal cell types (D1,D2,A2a, Chat)

• D1 ablation: reduced opioid (morphine and OXY) locomotor and sensitization. No effect on IVSA

• A2a deletion (subset of D2 neurons): increased opioid locomotion, sensitization, and seeking during extinction

• ChaT ablation: decreased morphine loco, no effect on OXY loco. Increased seeking during extinction

• D2 deletion: no effect.

• Comprehensive introduction and thoughtful discussion. Covers limitations, alternate explanations, etc.

• Beautiful paper, tour de force. Suggestions for improvement are provide below.

Abstract

• What does "striatal centric" refer to in the title? What does the centric mean?

• Target MOR in D1, D2, A2a, and ChAT

• What KO MOR allele are they using to induce cell type-specific KO?

• D1 MOR deletion: removed opioid-induced hyperlocomotion

• D2 & ChAT MOR deletion: no effect

• D2 A2a deletion: enhanced locomotion (opioid-specific or all?)

• Opioid-induced (what drug)?

• Doesn‘t say in the abstract how MOR was deleted within these cells specifically within the striatum? Cre-loxP methodology should be mentioned in the Abstract

Methods

• What does 50/50 B6/129 background mean for the floxed MOR Mice? How is that maintained? Additional details on the breeding scheme for maintaining these lines should be provided. What is the specific 129 substrain that comprises the mixed background of the floxed MOR mice?

• It is stated that the floxed genotype is homozygous but that Cre recombinase was inherited on either 1 allele (D1,D2,A2A) or 2 alleles (ChAT). Wouldn’t a consistent allelic state of Cre across all four cell types of interest make the results more interpretable? One could invision that 2 copies vs one copy of Cre could be more effective at excising floxed MOR which in turn, could further alter behavior.

• Does 100% C57BL/6J for the Cre lines refer to backcrossing for > 10 generations or were they actually generated from the start on a pure C57BL/6J background?

• Sample sizes are small, no power analysis is provided, no mention of Sex as a Biological Variable (SABV), even though both females and males were run. Lack of power could lead to both false positive and false negative results with mixed sexes, especially if the sexes are unbalanced but also if there is a real Sex x Genotype interaction. Females are known to show a greater locomotor stimulant response to opioids. At the very least, specific Ns per sex per genotype per treatment should be reported. Ideally, Sex would be included in the statistical model either as a factor in an ANOVA model or as a covariate in a LMM to account for variance due to Sex.

Figure Legends

• "Day 1", "Day 3" terminology was a bit confusing at first pass. What about Day 2? Also, why not report the locomotor habituation data prior to the saline/opioid injections? (Figures 2, 3). This could inform the interpretation of the opioid response, regarding whether it is selective or non-selective due to lack of habituation/persistent response to novelty.

• The time course of the lever presses during IVSA is informative and interesting. (panels i-n). but what are the "three specific days of the IVSA protocol" though? I was able to discern that at least two of these are Day 1 and Day 3 of extinction from the subsequent text in the figure legend but this should be stated earlier in the legend. Is it simply LPs and RNFS during Day 1 (i,j), Day 2 (k,l), and Day 3 (m,n) of extinction?

Figures (recommendations).

Generally speaking, there is room to make these figures more informative and easier for the reader to digest independent from flipping back and forth between the legend.

• Fig.2b: day 1 and day 3 could be specified with "oxy"

• Fig.3a-3c: could add "oxy" and "morphine" underneath the doses to specify which drug is which

• Fig.3d-3h: please indicate the particular cell type targeted.

• Fig.3i-3l: would be helpful to add something like "acute oxy day 1", "sensitized oxy day 3" , "acute morphine day 1", and "sensitized morphine day 3’

• It would also be helpful to indicate the doses of morphine (15 mg/kg) and oxy (10 mg/kg) used for inducing sensitization.

• Figure 4a-4g: "number" y-axis label: could be more informative (# lever presses? # nose pokes?)

• Figure 4h: Same. "Percent" what? Consider making this label more informative.

Reviewer #2:

The authors dissected the contribution of deletion of the mu opioid receptor (MOR) in A2a-, ChAT-, D2-, and D1-positive neurons to opioid-induced locomotion and oxycodone and morphine self-administration. The authors evaluated differences between each line following extinction. Further dissection of the contribution MORs in specific populations of neurons is useful to the field, but significant revision of the figures is necessary. Furthermore, interpretation of the results hinges on the focus of striatal neurons, but the method of deletion can impact MORs anywhere there is cre expression. The authors need to further explain the consequences of this method on the data and interpretation of the results.

Major Concerns

1) The fundamental crux of the study relies on the co-expression of floxed MOR and cre expression in cells that are positive for A2a, D1, D2, and CHAT. The authors need to further discuss the co-expression of MORs and each receptor outside the striatum. There are several other brain regions where both MORs and these receptors are expressed. How does this impact the behaviors that the authors are measuring and the animals’ response to drug? Does this line also lack MOR expression in the peripheral nervous system? It may be beneficial to make a figure that shows the brain-wide distribution of each cell type (A2a, D1, D2, CHAT) and how it overlaps with MORs. Erbs et al. extensively described MOR expression (PMID 24623156). How could this change the interpretation of the results?

2) It is obvious that the authors have done a lot of work, and they should be commended for displaying individuals on each graph, but the figures are very difficult to understand. There are many missing labels (doses, drug concentrations, etc.). It would be beneficial to simplify the data and move alternative measures and graphs to supplementary material or combine into additional figures.

a. For example, in Fig. 2a, A2AflMOR animals exhibit the greatest variability in distance traveled. Were these data assessed for outliers and compared with expression levels to ensure that knockout was achieved in these animals? I am also confused by the labeling of "A" and "B" in the bar graphs for distance traveled. Why are they not all oxycodone or morphine? Are some of each? For Fig. 3c, it would be helpful to label on the graph that you are testing cocaine and the dose (there is no label on the X-axis).

b. Fig. 4 shows axes that are labeled simply "number." What does this mean? Do the error bars show the SEM or SD? It is also unclear which graphs represent which measures without referring to the figure legend. A title for each graph would make them much easier to understand.

c. In Fig. 4M and N, the authors show the number of active lever presses (4M; this reviewer assumes this is despite reward) and the cumulative reinforcers ("RNFS"; 4N). How is it that A2aflMOR animals have much higher number of active lever presses, but ChAT animals have a significantly higher number of RNFS? There is no discussion of the implication of higher active lever presses without reward and what this could mean in the context of the experiment for each line.

d. The RNA scope data are difficult to interpret. There is also no A2a expression overlap with the MOR data, but there are two lines of panels for CHAT (with a repeat of flMOR). Given that the authors posit that A2a is deleted from a subset of D2 neurons, the RNAScope of A2a should be included and would strengthen this hypothesis. Insets of deleted and non-deleted cell types would be useful. Furthermore, there appears to be four ChAT-positive neurons in the bottom panel, two of which are positive for MOR following deletion. How do the authors account for this, given that a cell was counted positive with three foci present within the cell?

Minor Concerns

3) I understand the need to shorten and abbreviate some words, but some sentences are almost completely acronyms and abbreviations, making the text very difficult to read. The authors need to refrain from using so many abbreviations. Just spell them out and only retain the most common abbreviations.
